# Complications of patients with bone tumors treated with carbon-fiber plates: an international multicenter study

**DOI:** 10.1038/s41598-022-23519-9

**Published:** 2022-11-08

**Authors:** Carbon-Fiber International Collaboration Initiative Research Group, Zeger Rijs, Amber Weekhout, Santiago A. Lozano-Calderon, Olivier Q. Groot, Emily Berner, Nelson Merchan, Caleb M. Yeung, Vsania Oliveira, Giuseppe Bianchi, Eric Staals, Debora Lana, Davide Donati, Ortal Segal, Stefano Marone, Raimondo Piana, Simone De Meo, Pietro Pellegrino, Nicola Ratto, Carmine Zoccali, Maurizio Scorianz, Cecilia Tomai, Guido Scoccianti, Domenico Andrea Campanacci, Lorenzo Andreani, Silvia de Franco, Michele Boffano, Thomas Cosker, Varunprasanth Sethurajah, Manuel Peleteiro Pensado, Irene Barrientos Ruiz, Esperanza Holgado Moreno, Eduardo Jose Ortiz-Cruz, Michiel van de Sande

**Affiliations:** 1 Department of Orthopaedics, Leiden University Medical Center, Leiden, The Netherlands; 2 Department of Orthopaedics, Massachusetts General Hospital—Harvard Medical School, Boston, USA; 3 Department of Orthopaedics, Centro Hospitalar Universitário Do Porto, Porto, Portugal; 4 Orthopaedic Oncology Unit, IRCCS Istituto Ortopedico Rizzoli, Bologna, Italy; 5 Department of Orthopaedics, Tel Aviv Sourasky Medical Center, Tel Aviv, Israel; 6 Department of Orthopaedic Oncologic Surgery, Centro Traumatologico Ortopedico Turin, Turin, Italy; 7 Oncological Orthopaedics Unit, Regina Elena National Cancer Institute, Rome, Italy; 8 Department of Orthopaedic Oncology and Reconstructive Surgery, Azienda Ospedaliero-Universitaria Careggi, Florence, Italy; 9 Department of Orthopaedic and Trauma Surgery, University Hospital of Pisa, Pisa, Italy; 10 Department of Orthopaedic Oncology and Reconstructive Surgery, Regina Margherita Children’s Hospital, Turin, Italy; 11 Department of Orthopaedics, Nuffield Orthopaedic Centre, Oxford, England; 12 Department of Orthopaedics, Hospital Universitario La Paz, Madrid, Spain; 13 Department of Orthopaedics, MD Anderson Cancer Center Madrid, Madrid, Spain

**Keywords:** Surgical oncology, Bone cancer

## Abstract

Carbon-fiber (CF) plates are a promising alternative to metal plates. However, reported experience in orthopaedic oncology remains limited. The aim of this study was to identify complications of patients with bone tumors treated with CF plates. Between February 2015 and May 2021, 13 centers retrospectively registered patients with bone tumors that were reconstructed using CF plates. Complications were identified, and timing and etiology of complications were noted. Similar complications were tabulated and classified based on mechanical, non-mechanical and paediatric complications. Mechanical complications included: (1) aseptic loosening or graft-host non-union, and (2) structural complications. Non-mechanical complications included: (3) soft tissue complications, (4) infection and (5) tumor progression. Specific paediatric complications included (6) growth arrest resulting in longitudinal or angular deformity. Ninety-six patients were included with a median follow-up time of 35 months. In total, 22 (23%) patients had complications. Mechanical complications included: 1 (1%) aseptic loosening, 2 (2%) non-unions, and 7 (7%) structural complications. Non-mechanical complications included 1 (1%) soft tissue complication, 4 (4%) infections and 5 (5%) tumor progressions. Paediatric complications occurred in 2 (2%) patients. This study suggests CF plates are safe to use in demanding reconstructions after bone tumor resections, presenting a seemingly low complication profile.

## Introduction

Metal has been the foundation of orthopaedic implants. Advantages include high strength and stiffness, ease of machining, and low cost^1^. Many metals also offer good ductility allowing them to be manually bent intraoperatively to match the surface anatomy of the bone or reconstruction^1,2^. However, a major disadvantage for the oncological patient is its radiodensity which causes metal artifacts on radiographic imaging. This precludes accurate radiographic visualization for oncological follow-up or bone union and impedes precise radiation planning^3,4^. Besides, the stiffness of metal (200 gigapascal [GPa] for stainless steel and 110 GPa for titanium) is much higher than the human cortical bone (12 GPa) which may shield the underlying bone from stress and can lead to reduced bone quality^5,6^. Other disadvantages of metal implants include limited fatigue life, potential for generation of wear debris, cold welding, and corrosion^1,6,7^. Consequently, there is a demand for improved orthopaedic implants.

**Figure 1:**
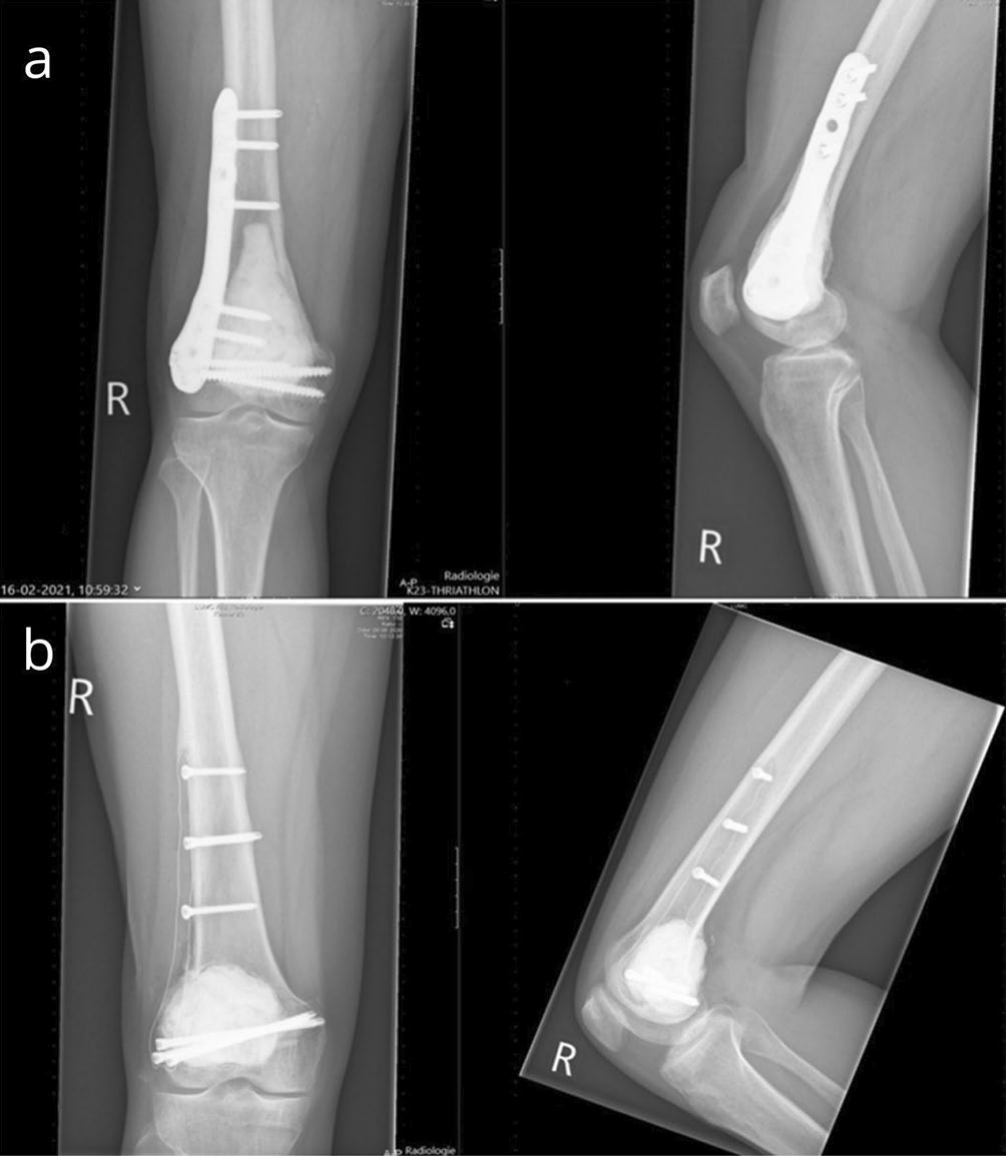
Giant cell tumor in the right distal femur treated with bone cement during curettage using a metal plate (**a**) and a carbon-fiber plate (**b**).

Carbon-fiber (CF), reinforced with polyetheretherketone, is one of the promising innovative implant materials in the field of orthopaedic oncology. CF plates are increasingly used and offer several benefits compared with metal. First, CF’s radiolucency allows for precise radiation planning and better radiologic visualization of local tumor recurrences and bone healing, thereby facilitating improved postoperative follow-up and surveillance for oncological patients (Fig. 1)^8–11^. Second, the modulus of elasticity of CF (13 GPa) is closer to cortical bone (12 GPa)^12^. Third, CF has the capability to withstand prolonged fatigue strength compared with current metal plates^12^. Therefore, biomechanical properties of CF should theoretically enhance bone healing and reduce complication risks. Lastly, other material-specific advantages include easier implant removal due to the metallic screws and polymeric plate (no cold welding) and the lack of metallic allergy^13^.

Despite these advantages, CF composites have shown brittle failure in tension and flexural tests^14^. When this occurs, the material breaks into multiple solid fragments instead of deforming or straining under load. This was reported, under supra-physiological load in vitro*,* in 2 out of 12 simulated comminute distal fibula fractures treated with CF plates^15^. Additionally, intraoperative plate breakage occurred while inserting a screw to obtain fracture reduction by tightening the plate to the bone in 3 out of 78 proximal humerus CF plates in non-oncological patients^16^. Intraoperative plate breakage was also reported in 5 out of 110 distal radius fractures treated with volar CF plates^13^. Regarding oncological patients, one CF plate failure was reported 4 months after implantation in a 75-year-old patient with lymphoma, while the postoperative course of 2 CF plates was uneventful (77-year-old male with prostatic carcinoma metastasis in the humerus and 17-year-old patient with an intraosseous schwannoma in the tibia with 6 and 8 months of follow-up, respectively)^17,18^.

Although CF plates are increasingly used in fracture care, reported experience in orthopaedic oncology remains limited. Therefore, the purpose of this study was to identify complications of patients with bone tumors treated with CF plates.

## Materials and methods

**Figure 2:**
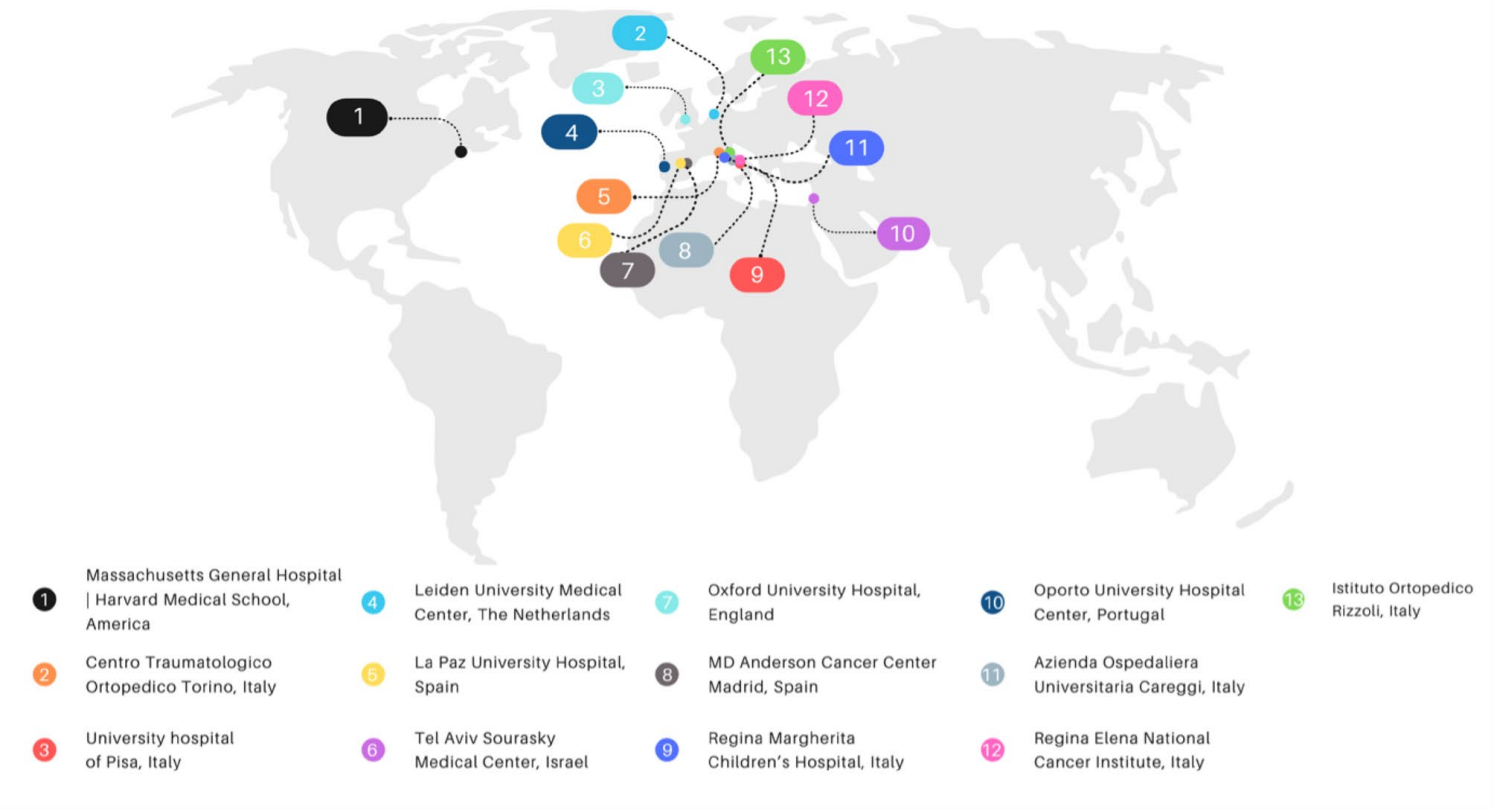
World map showing all 13 participating centers.

This retrospective study is based on the experience of the “Carbon-Fiber International Collaboration Initiative” research group which included 13 large academic and non-academic hospitals from Europe, the Middle East, the United Kingdom, and the United States of America (Fig. 2). The study protocol was approved by the ethics committee Leiden (coordinating center), and each of the participating centers’ institutional review board. Data exchange agreements were signed before patient inclusion started. Due to the observational nature of the study and with the aim to assess quality of the CF implants used, further ethical approval including informed consent was waived by the Medical Ethics Review Committee Leiden Den Haag Delft, reference G20.103. Data was collected through a centralized online Castor electronic data capture database^19^. The coordinating center (Leiden University Medical Center) had access to all data entered in Castor. All methods were performed in accordance with relevant guidelines and regulations.

### Participants and treatment details

**Figure 3:**
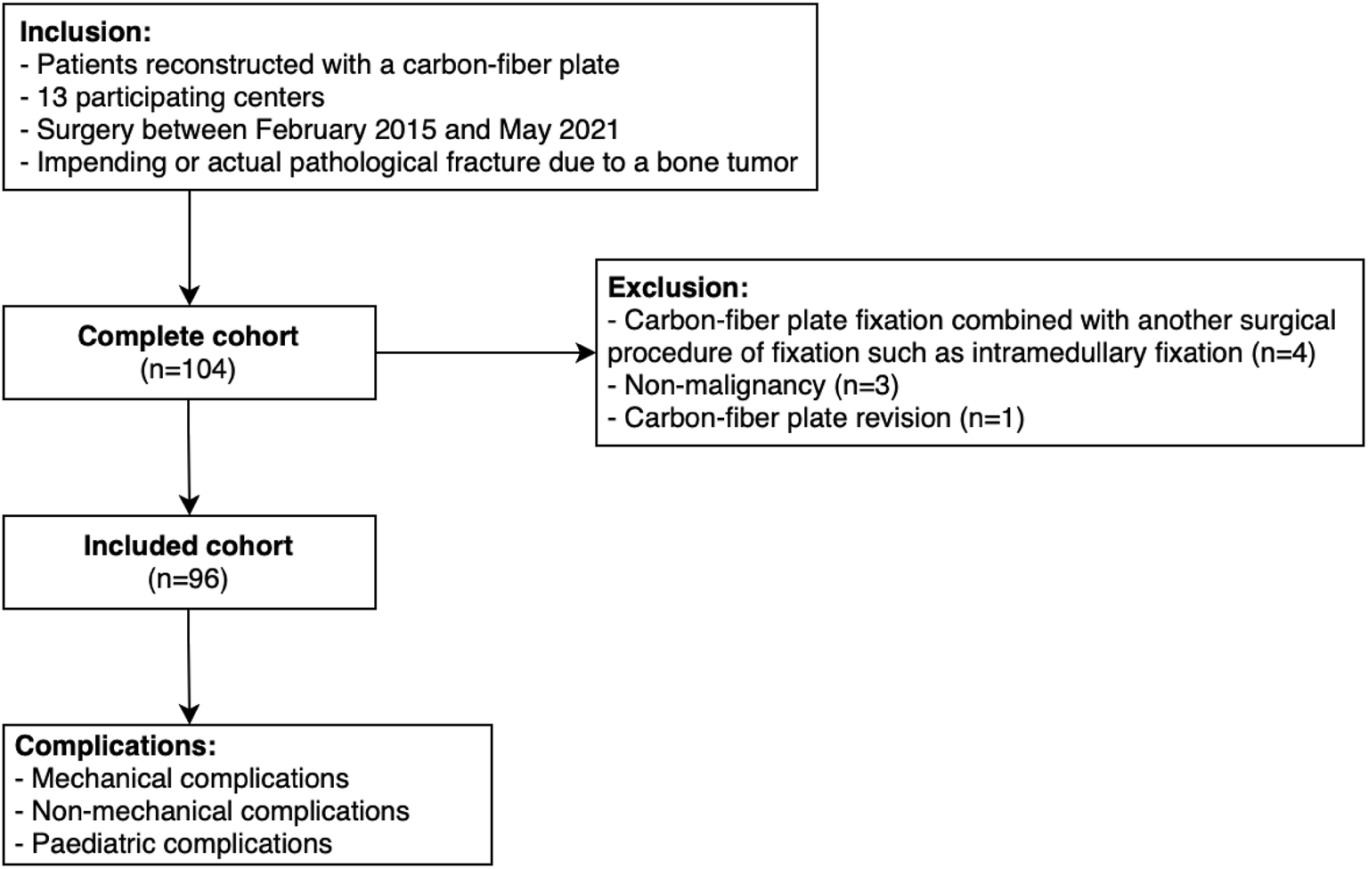
Flow diagram illustrating patient selection and outcomes.

Between February 2015 and May 2021, all sequential patients who received a CF plate were retrospectively included by each participating center without age restriction. Patients who received more than one CF plate during the same surgery were also included. Patients were excluded in case of (1) a combination of CF plate fixation with another surgical procedure of fixation such as intramedullary fixation, (2) non-malignancy, and (3) CF plate revisions. Only the first surgery was included if a patient had more than one qualifying surgery during the study period (Fig. 3).

**Figure 4:**
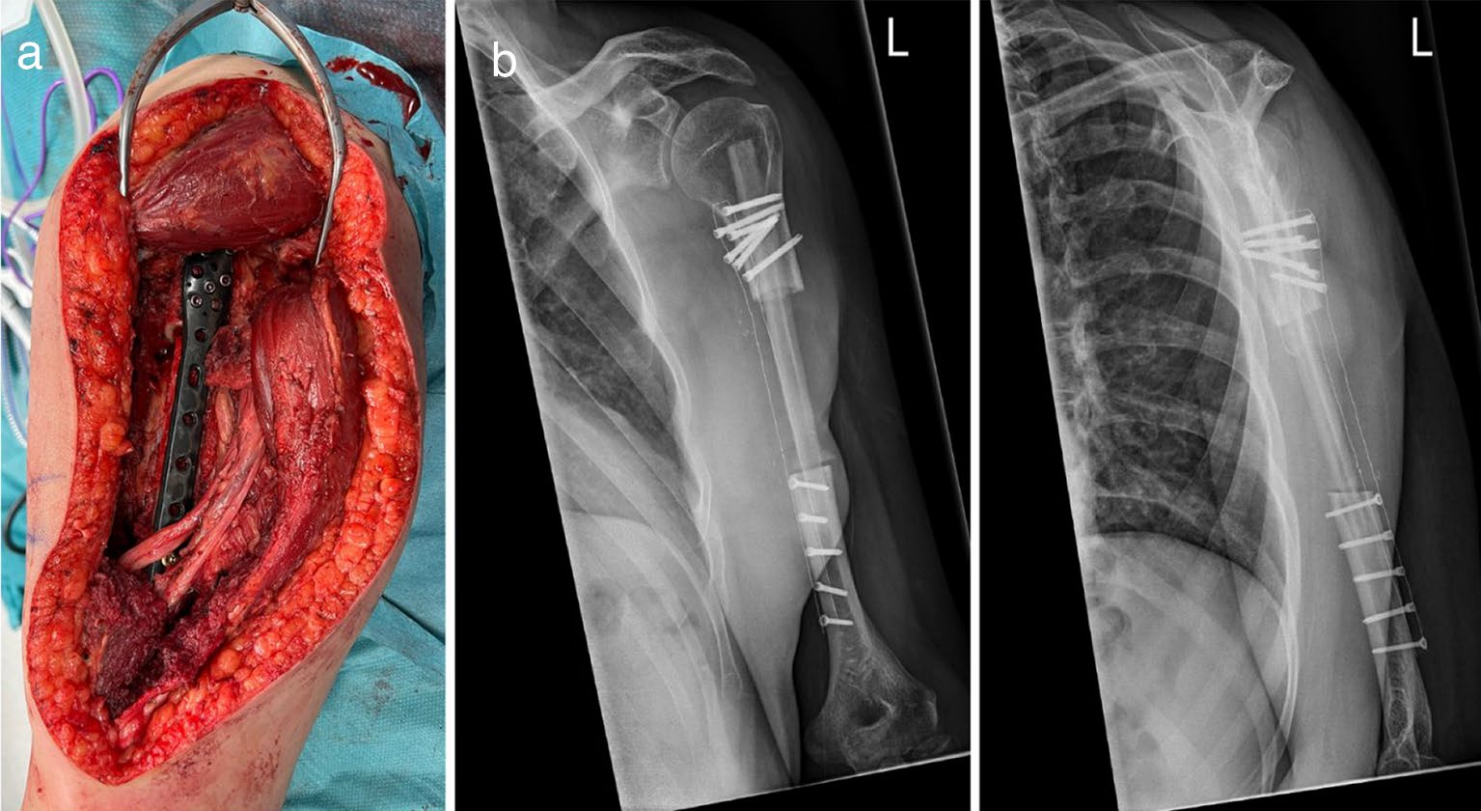
Intraoperative picture after midshaft resection of the left humerus and partial resection of the triceps muscle due to an Ewing Sarcoma. Reconstruction was performed with a free vascularized fibula graft and a carbon-fiber humerus plate (**a**). Postoperative anteroposterior X-rays of the same patient (**b**).

The choice of treatment was made by shared decision making between the patient and surgeon. In general, surgery was recommended to oncological patients with impending or actual pathological fractures, mechanical axial loading pain, and no response to radiation therapy or oral narcotic pain medication. The choice of using a CF plate instead of a conventional metal plate was made by the operating surgeon. Length of the plates would not have been different between CF plates or conventional metal plates. Good candidates for CF plates were patients with standard anatomy because CF plates cannot easily be bend manually to match surface anatomy of individual bones. Therefore, surgeons must ensure good implant fit preoperatively. During this study, patients were treated with various FDA approved and CE marked CF plates with locking screw options (manufactured by CarboFix Orthopaedics; Herzeliya, Israel) (Fig. 4). The surgical procedure, including positioning of the patient and surgical approach, depended on the surgeon’s experience and preference. All oncological patients were clinically and radiographically evaluated postoperatively after 6-months, 1-year, and 2-years. Subsequent follow-up visits with radiographic evaluation were dependent on the patient’s oncological status, and additional visits took place if needed. Patients were cleared for radiation therapy or chemotherapy 7–10 days after the surgical procedure and all patients adhered to weight bearing as tolerated after completion of surgery. The rate of loss to follow-up was 1% (1/96) at 6-months, 2% (2/96) at 1-year, and 5% (5/96) at 2-year. Five patients were lost to follow-up due to death of disease during the standard 2-year follow-up period. Follow-up was verified until July 8th, 2022.

### Variables and outcome measures

The following clinical variables were registered: sex; age; body mass index (BMI); smoking status (non-smoker was defined as stopped at least 6 months ago); American Society of Anesthesiologists (ASA) score; diagnosis/indication; tumor grade; preoperative chemotherapy; preoperative radiotherapy; postoperative chemotherapy within 6 months of surgery; postoperative radiotherapy within 6 months of surgery; date of surgery; surgical side; pathological fracture; location of surgery; location of bone; use of autograft, allograft, or cement; surgical margin; and type of CF plate.

**Figure 5:**
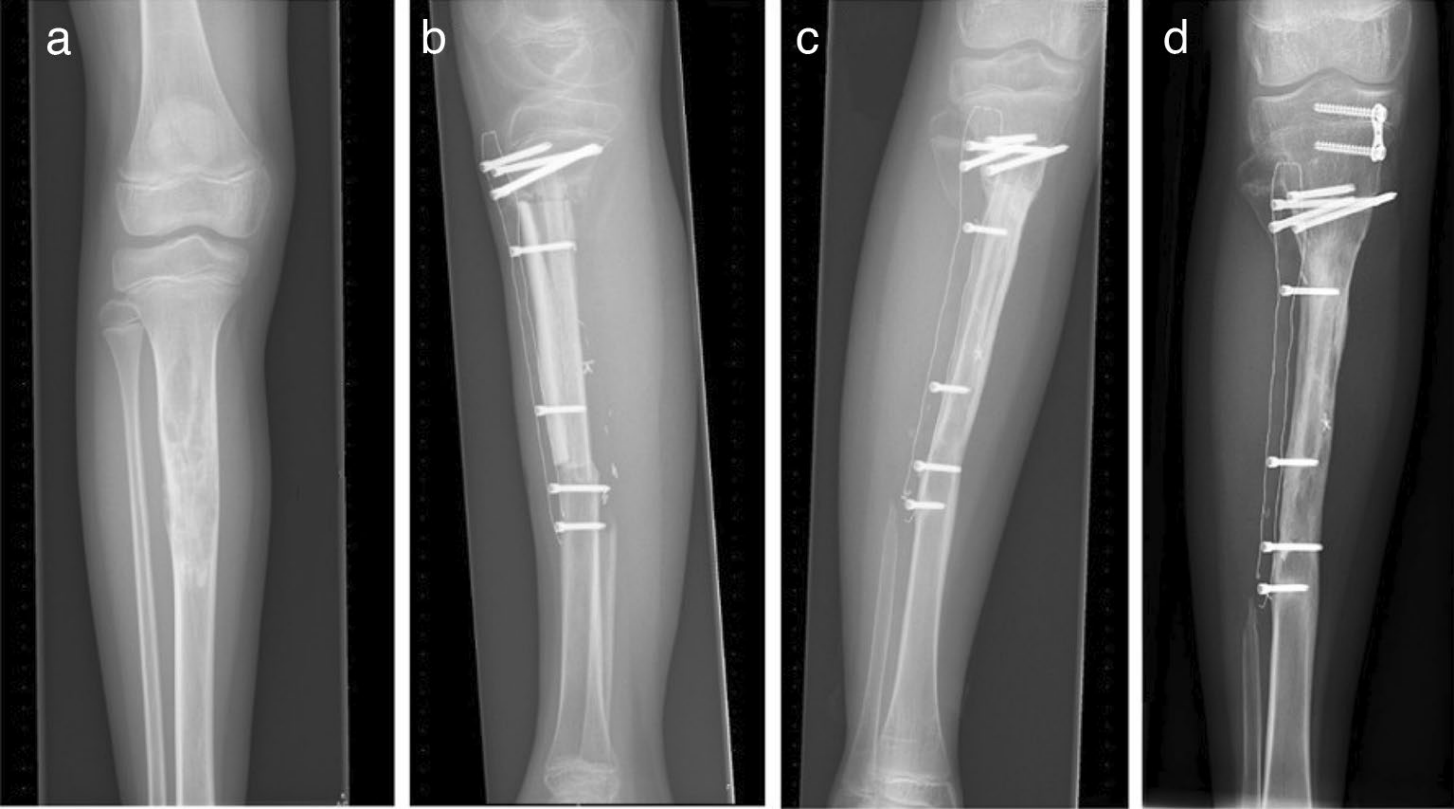
Adamantinoma in the proximal tibia of a 10-year-old girl (**a**). Status after resection proximal tibia and reconstruction with humerus allograft, fibula transfer and carbon-fiber plate. Allograft-host junction healing at (**b**) 6 months, (**c**) 1-year, and (**d**) 2-years postoperatively. Additional surgery was performed to treat the valgus leg axis with an eight-plate 21 months after initial surgery (**d**).

Patients who had complications were identified, and timing and etiology of complications were noted. Similar complications were tabulated and classified based on mechanical, non-mechanical and paediatric complications. Mechanical complications included: (1) aseptic loosening or graft-host non-union in case of a biological reconstruction, and (2) structural complications such as periprosthetic fracture or plate breakage. Radiologic presence of mature bridging bone at graft-host junction site was considered bony union (Fig. 5). Any patient failing to show bony union 1-year postoperatively or patients that required additional surgery to achieve healing was defined as having a non-union. Non-mechanical complications included: (3) soft tissue complications such as wound dehiscence, (4) infection and (5) tumor progression. Specific paediatric complications included (6) growth arrest resulting in longitudinal or angular deformity.

### Statistical methods

Descriptive statistics were performed using SPSS v.24 (IBM Corp., Armonk, NY, USA). Baseline characteristics and surgical variables were shown using frequencies (percentages for categorical variables) and medians (interquartile ranges [IQR’s] for continuous variables as they were not normally distributed based on histogram inspection).

## Results

In total, 96 patients of which 59 female (61%) with a median age of 43 years (IQR; 19–54) were included with a median follow-up of 35 months (IQR; 21–49). The three most common indications included atypical cartilaginous tumors (34%), benign primary bone lesions (28%), and osteosarcomas (12%). Most lesions were located in the femur (70%), followed by the tibia (15%) and humerus (14%). The majority of surgical margins were intralesional (60%), followed by wide margins (19%), marginal margins (13%), no resection (5%), and not reported (3%). In total, 11 (12%) patients received an autograft, 43 (45%) received an allograft, and 43 (45%) received cement. Three (3%) patients received a diaphyseal and metaphyseal CF plate combined during the same surgical procedure (Table 1).

**Table 1:** Demographic features of included patients treated with carbon-fiber plates (n = 96).

Baseline characteristics	n (%)
Female	59 (61%)
Age (years; median with IQR)	43 (19–54)
BMI (kg/m^2^;^a^ median with IQR)	24 (20–27)
Smoking status^a^	17 (18%)
**ASA score**^**a**^	
1–2	81 (84%)
3–4	12 (13%)
**Diagnosis/indication**	
Atypical cartilaginous tumors	33 (34%)
Benign primary bone lesions	27 (28%)
Osteosarcoma	11 (12%)
Adamantinoma	10 (10%)
Metastasis	7 (7%)
Multiple myeloma	3 (3%)
Soft-tissue sarcoma with bone invasion	3 (3%)
Ewing Sarcoma	2 (2%)
**Tumor grade**^**a**^** (excluded benign primary bone lesions**^**b**^**)**	
Low	44 (46%)
High	24 (25%)
Preoperative chemotherapy	17 (18%)
Preoperative radiotherapy to surgery site	4 (4%)
Postoperative chemotherapy	18 (19%)
Postoperative radiotherapy	7 (7%)
**Surgical variables**	
Surgical side	
Left	61 (64%)
Right	35 (36%)
Pathological fracture	14 (15%)
**Location of surgery**	
Femur	67 (70%)
Tibia	14 (15%)
Humerus	13 (14%)
Radius	2 (2%)
**Location of bone**	
Diaphyseal	39 (41%)
Metaphyseal	38 (40%)
Epiphyseal	6 (6%)
Diaphyseal and Metaphysial combined^c^	7 (7%)
Metadiaphyseal and Epiphyseal combined	6 (6%)
Cement	43 (45%)
Allograft	43 (45%)
Autograft	11 (12%)
**Type of carbon-fiber plate**	
Femoral condyle plate	42 (44%)
Diaphyseal broad femur plate	22 (23%)
Femoral condyle and diaphyseal broad plate combined^d^	3 (3%)
Diaphyseal narrow femur plate	4 (4%)
Proximal humerus plate	23 (24%)
Distal radius plate	2 (2%)
**Surgical margin**^**a**^	
Intralesional	58 (60%)
Marginal	12 (13%)
Wide	18 (19%)
No resection	5 (5%)

*IQR* = interquartile range, *ASA* = American society of anaesthesiologists, *BMI* = body mass index.

^a^Missing data was present in BMI for 14/96 (15%); smoking status 13/96 (13%); ASA-score 3/96 (3%); Tumor grade 1/96 (1%); and surgical margin 3/96 (3%).

^b^Stage (latent, active, aggressive) of benign bone lesions was not reported.

^c^Three patients received a diaphyseal and metaphyseal plate combined during the same surgical procedure. Therefore, a total of 99 plates were placed in 96 patients.

^d^Type of carbon-fiber plate can be different to “location of surgery”. For example, proximal humerus plates were used for lesions in the tibia (predominantly children) because carbon-fiber tibia plates were not yet available (FDA approved since October 2020).

**Figure 6:**
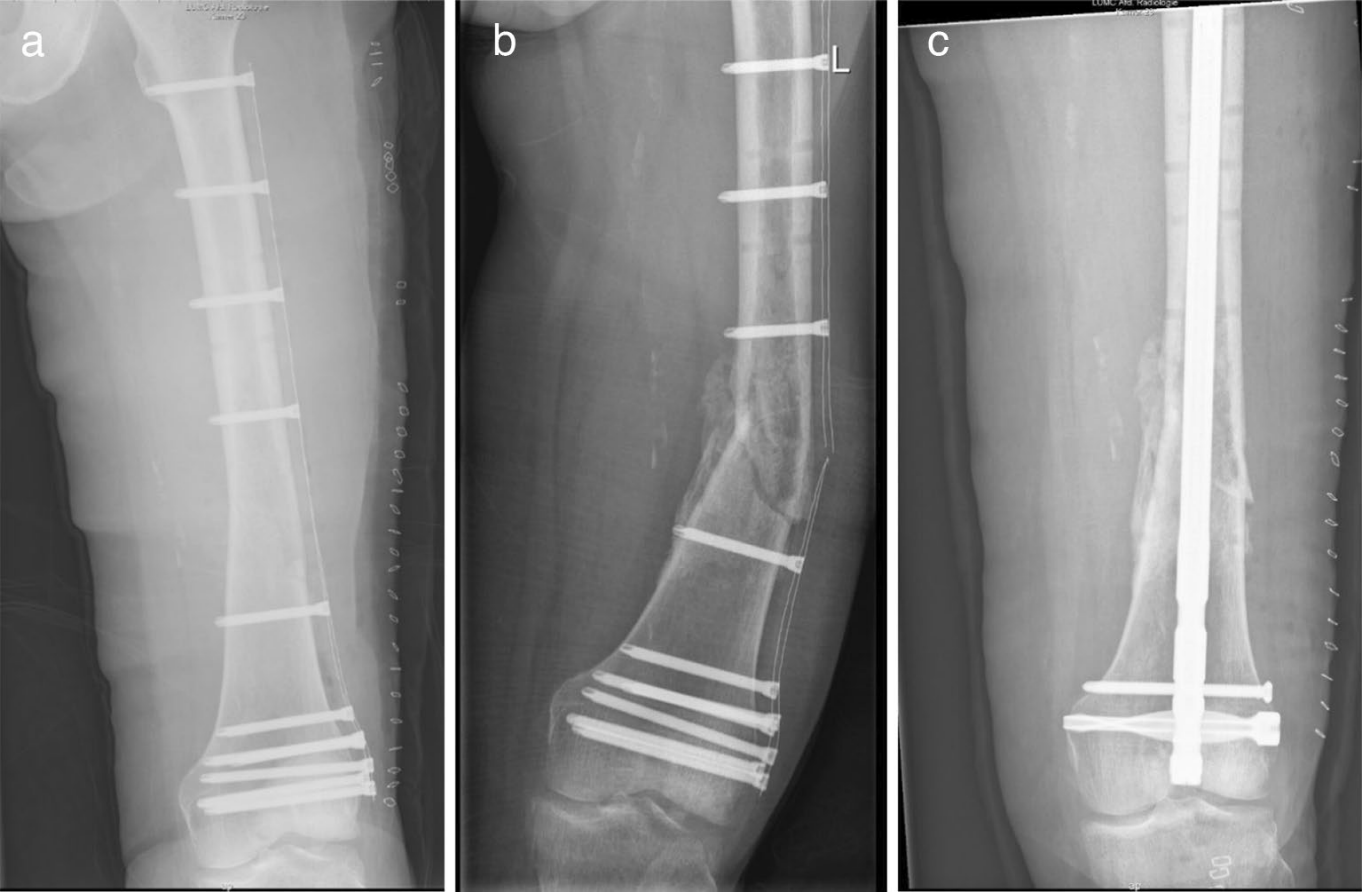
Carbon-fiber plate after pathological fracture of the left distal femur due to a diffuse large B-cell lymphoma (**a**). Plate breakage, exactly at the location of the pathological fracture at 5 months after surgery (**b**). Status after revision with a conventional retrograde femoral nail (**c**). Pseudoarthrosis remained, and this patient died of disease 1-year after the carbon-fiber plate revision with a conventional retrograde femoral nail. In general, an intramedullary osteosynthesis of lower extremity pathological fractures is preferred because steel plates are expected to break when fracture healing is not achieved.

In total, 22 (23%) patients endured complications (Table 2). Mechanical complications included 1 patient with aseptic loosening of the CF plate after 20 months, and 2 non-unions after biological reconstruction with an allograft (20 and 28 months postoperative). Structural complications occurred in 7 patients. These complications included 2 periprosthetic fractures (1 and 3 months postoperative), 1 traumatic proximal humerus plate breakage (14-year-old male fell off his bike 28 months after surgery) and 2 femoral condyle plate breakages without clear trauma (75-year-old female 5 months postoperative “stood up from bed”, and 19-year-old male 2 months postoperative “while getting dressed”) (Fig. 6). In these cases, full weight bearing with incomplete bone healing and malalignment of the reconstruction was considered the cause of plate breakage. Further structural complications included 1 screw breakage (9 months postoperative), and 1 screw backing out (2 months postoperative). Non-mechanical complications included 1 patient with wound dehiscence within a month after surgery (this patient received preoperative radiotherapy with a total dose of 50 Gy), 4 infections (less than a month, 1, 6 and 10 months postoperative); and 5 tumor progressions which lead to a transfemoral amputation in one case (5, 7, 17, 20 and 31 months postoperative). Specific paediatric complications occurred in 2 patients in which eight-plates were placed to treat valgus deformations (21 and 28 months postoperative). Interestingly, almost all mechanical complications, except for a traumatic humerus plate breakage, occurred in CF plates placed in the lower extremity. Non-mechanical complications were equally distributed between the upper- and lower extremity, and paediatric complications occurred in the lower extremity. Besides, 5 of the CF plates were removed due to irritation/pain at the site of the implant after complete bone healing (after 12, 20, 21, 36, and 40 months).

**Table 2:** Complications of patients with bone tumors treated with carbon-fiber plates.

	Time to complication (in months)	Etiology
**Mechanical complications**		
(1) Aseptic loosening or non-union in case of a biological reconstruction	20	**1 (1%)** Aseptic loosening for which the CF plate was removed
	20, 28	**2 (2%)** Non-unions after reconstruction with an allograft. CF plate still in situ for both cases
(2) Structural complications	1, 3	**2 (2%)** Periprosthetic fractures without clear mechanism. CF plate was still in situ for both, one patient died
	28	**1 (1%)** One traumatic plate breakage (14-year-old male fell off his bike)
	2, 5	**2 (2%)** Plate breakages without clear trauma. Full weight bearing with incomplete bone healing and malalignment of the reconstruction identified as cause of plate breakage. CF plates were removed
	9	**1 (1%)** Screw breakage. CF plate still in situ
	2	**1 (1%)** Screw backed out and was removed without any difficulty. CF plate remained in situ
**Non-mechanical complications**		
(3) Soft tissue complications	0	**1 (1%)** Wound dehiscence after preoperative radiotherapy (total dose 50 Gy) requiring irrigation and removal of the CF plate
(4) Infection	0, 1, 6, 10	**4 (4%)** infections: Two infections resulted in removal of the CF plate. The other two patients were successfully treated with debridement and long-lasting antibiotics
(5) Tumor progression	5, 7, 17, 20, 31	**5 (5%)** Tumor progression: Two local recurrences resulted in CF plate removal, one of those patients was treated with a transfemoral amputation. Two other patients died of disease with the CF plate in situ and one local recurrence was successfully treated with thermoablation
**Paediatric complications**		
(6) Growth arrest resulting in longitudinal or angular deformity	21, 28	**2 (2%)** Angular deformities. Both patients were treated with an eight-plate on the medial side of the proximal tibia for a valgus leg axis after resection of an adamantinoma. CF plates are still in situ

*CF* = carbon-fiber.

## Discussion

Although CF plates are already used worldwide, reported experience in orthopaedic oncology remains limited. Describing complications of patients with bone tumors treated with CF plates offers valuable information for orthopaedic oncologists that may want to use CF plates. This international multicenter study evaluated 96 patients with bone tumors treated with CF plates. During the study period with a median follow-up of 35 months (IQR; 21–49), 22 (23%) patients were reported to have complications, which suggests CF plates are safe to use in patients with bone tumors that often require demanding reconstructions. Particularly the low percentage of nun-unions (2%) with high percentages of biological reconstructions (12% autograft and 45% allograft) are promising. To date, this is the largest CF plates cohort reporting on complications in an oncologic population.

The major disadvantage of CF plates is its inability to be manually bent to match surface anatomy of individual bones. Therefore, surgeons must ensure good implant fit preoperatively. CF plates could not be used during all reconstructive surgeries due to unique anatomy or complex mechanical problems. For some complex cases, conventional metal implants that can be bend, customizable orthopaedic implants, or patient specific implants that can better match the reconstructed anatomy may be preferred^20^. However, patient specific implants are a time-consuming alternative, and it is still uncertain whether the theoretical biomechanical advantages carry true advantages in surgical outcomes when compared to standard procedures^20,21^. Secondly, while CF plate’s radiolucency is beneficial for postoperative radiological imaging, determining the optimal plate position can be challenging. Thirdly, production costs and availability could be another disadvantage. However, CF reinforced composites have become more competitive and are widely used across industries like aerospace, wind energy, and automotive^22^. As a result, production costs have decreased, and the costs of CF plates are currently competitive with conventional metal plates.

Although study groups and surgical procedure are not always comparable, it may be noted that our study provides relatively low non-union rates (2%), even with a high percentage of biological reconstructions (12% autograft and 45% allograft). Wisanuyotin et al. reported 30% nonunion (mean time to union of 9.8 ± 2.9 months) for nonvascularized autograft (NA), and 32% nonunion (mean time to union of 11.5 ± 2.8 months) for allografts after resection and reconstruction of primary bone tumors^23^. In addition, Buecker et al. reported that locking plates for allograft-host junction fixation were associated with improved union rates compared with standard plates (75% union at an average of 13 months versus 56% at an average of 14 months, respectively)^24^. Moreover, the total rate of CF plate complications (23%) was low compared to conventional metal plate studies (complication range 42–76% with follow-up range of 35–112 months)^25–28^. When comparing our results with CF plates placed for trauma patients, we reported 12 (18%) CF femoral plate failures while Byun et al. and Mitchell et al. reported none (0%) and 1 (9%) failure in respectively 10 and 11 patients treated with CF femoral plates^29,30^. Although the number of failures is currently too small to identify risk factors for plate complications, higher complication rates can be expected with more extensive treatment such as chemo- and/or radiotherapy and more complex surgery with auto-/allografts^27^.

This study has several limitations. First, this remains a single-arm retrospective international multicenter study with inherent limitations associated with such a study design, including the lack of a comparison group and reliance on chart abstraction. As a result, our study was prone to selection bias. However, participating centers were asked to sequentially include patients according to a standard inclusion protocol. Centers were regularly contacted by the coordinating center to elaborate on cases if there were any questions, ambiguities, or missing data. Nevertheless, the authors acknowledge that the most scientifically robust study design to assess the added value of CF plates is a randomized controlled trial with clinical-, radiological-, and functional outcomes as primary endpoints. However, patients with bone tumors in this group were heterogeneous in terms of baseline characteristics and surgical variables. Therefore, acquiring a matching control group would be difficult and we recommend propensity score matching as the next best step for future research. Second, performance bias could have occurred because the surgical procedure and postoperative management also depended on the surgeon’s experience and preference. Yet, no major differences in treatment outcomes between participating centers were observed.

## Conclusion

Carbon-fiber (CF) implants offer several material specific benefits compared to the more common metal implants. To assess safety of CF plates, we performed an international multicenter study describing all complications occurring in orthopedic oncology patients that were treated with CF plates. Low complication rates are reported, and complications originated mainly from disease progression or infection. Although based on a very heterogenous retrospective multicenter database our results suggests that orthopaedic oncologists may safely use CF plates in demanding reconstructions after bone tumor resections. However, studies of randomized or matched comparative nature are needed to assess the added clinical value of theoretical benefits of CF plates, such as precise radiation planning, improved bone healing, radiographic visualization of local recurrences and union.

## Data Availability

All the data related to the study are mentioned within the manuscript; however, the raw data are available with the corresponding author and will be provided upon a written request.
